# Some Thoughts Touching Excess of Mineral or Calcareous Matter in the System

**Published:** 1896-04

**Authors:** John C. Story

**Affiliations:** Dallas, Texas.


ARTICLE III.

### SOME THOUGHTS TOUCHING EXCESS OF MINERAL OR CALCAREOUS MATTER IN THE SYSTEM.

BY JOHN C. STORY, M. D., D. D. S., DALLAS, TEXAS.

Read before the Section on Oral and Dental Surgery, Nashville Academy of Medicine.

As our remarks in this paper deal almost entirely with the results of excessive deposits of mineral or calcareous matter in the system after the bony framework is complete (the most of which enter into the formation of bone), it will be well in the outset to consider for a brief space whence come these deposits, and then the results. Now, in all limestone regions (and here we find the conditions of which we are about to speak more prevalent than elsewhere, and more especially here in Texas, as this has been the field of our most extended observation, even the very air we breathe, and especially the water we drink. Add to this soda in some of its forms, which enters so largely into our various food preparations, with magnesia so often found in our drinking water, and then again add the not only useless, but harmful, salts of aluminum frequently found in our bread, and we have sufficient source for any amount of these deposits. These mineral substances, taken into the system in excess of the demand (even granting that they are ever demanded in that form), after the system or body is mature, constitute the tartar which we find deposited about the necks of the teeth, besides other stony formations to be noted further on.

Every observer is aware of the destructive influence of these deposits around the teeth—to the teeth themselves—causing more loss of these organs than all other sources combined, besides aiding and abetting other diseases with the vast amount of blood poisoning by reason of the pus generated about the necks of the teeth, from this irritant, and carried into the system through the digestive apparatus and by direct absorption.

But may we not also find in this excessive supply of earthy or mineral substances in the system the prime, yet hitherto occult, cause of some of our most fatal maladies? May we not here have the true cause attending fatal albuminuria or so-called Bright's disease? It must be admitted that every case of albuminuria is not Bright's disease, though every case of Bright's disease is albuminuria. Were it otherwise, few mothers would live to see their second baby. Happily, then, this is not the case. Albuminuria may be said to be a disease of nutrition (if disease at all), either excessive or perverted—in the pregnant woman evidently excessive, since the ruddy cheek, the increase of adipose tissue as shown in the rotundity of form, indicate the most perfect and robust health. An examination of the urine, however, at this time, will almost invariably indicate the presence of albumen. Then if albumen in the pregnant women comes from too much nutrition, may it not be the same in every other case? Now this albumen, being in excess, and not needed for use in building up the tissues, is instead taken by the various emunctories of the body and cast out with the refuse. Now may not this albumen in a system too well supplied with mineral salts, in its passage through the kidneys, the natural emunctory of the body for the mineral substances as the liver is for the vegetable, be there coagulated in the tubules, and form the little casts as we see them under the microscope, and are the pathognomonic signs of Bright's disease? And these coagula in turn furnish a nucleus for the deposit of the various earthy matters, forming little rough stones, it may be ever so minute, which by their presence set up ulcerative inflammation and develop true Bright's disease, destroying the structure of the kidney, and ultimately the life of the patient. It may be also that such a condition at the same time prevails in the great laboratory of the system, wherein are manufactured red blood corpuscles, since their constant diminution in the circulation is manifest from the increasing pallor or leaden hue as the disease progresses; and the almost uniformly sudden death of the patient, for, notwithstanding death is the inevitable result, it comes on almost always sooner than expected. The system having consumed the entire red blood supply, life goes out as does a lamp when the wick is exhausted. A reasonable foundation for these views is found in the fact that we have seen no case of Bright's disease in which there was not a large deposit of tartar about the teeth; and whoever saw a case in an individual brought up in the piny woods, and who had been raised on pure freestone water and a plain, frugal diet? In further confirmation, we note the rarity of this disease among children, and only in those of precocious development, and where the supply of calcareous matter had been largely in excess of the demand.

That we are led further to these conclusions comes from the fact that the remedies usually most efficacious in this fearfully fatal malady are found to be water absolutely pure or in which there is an excess of some acid, with an entire absence of the salts of lime, soda, etc., with a diet also free from these salts.

It might be given as a reason why the pregnant woman enjoys perfect immunity from this disease, notwithstanding her system is surcharged with albumen at this time, is the urgent demand for bone-making material for the upbuilding of the fœtus which she is growing in utero. And is it not a fact that calcareous deposits about the teeth are much lessened, if not entirely suspended, during this period?

The one great difficulty in the way of successful treatment of this disease lies in its insidiousness. Having its origin in too much health, it steals on the patient so gradually that he is often beyond the reach of hope or remedy before he becomes fully aware of his condition.

We wish to add, further, that these ideas, hypothetical as they are, have neither been bought, borrowed, nor stolen, since we have read nothing concerning this disease in thirty years, save, perhaps, the statements of the wonderful cures wrought by the various mineral waters in the advertisements of the wells and springs which produce them. But they are the results growing out of a study of the effects of these calcareous deposits in the diseases brought under our immediate observation by reason of the specialty of dentistry to which we have directed our attention for many years. Following the line of thought indicated by the known diseases produced by this evident surplus of calcareous matter, we have gone beyond the limit of our specialty and entered the field of general pathology with our speculations and arrived at the conclusions touching Bright's disease as given above. Going on then to amplify a little, if these conclusions are correct in regard to the etiology of this disease, have we not a key that will entirely shut the door to its ingress and, with many others, blot its name from the lists of incurables in our nosology?

To do this we have a task before us; but as physicians, philanthropists and true scientists, it is our duty to meet the condition with convincing arguments; arguments that will change entirely our dietetic system to the extent that calcareous matter in dangerous quantity will not be permitted to enter the system after the body is mature.

It is a well ascertained fact that all the mineral salts necessary to maintain in harmony the process of digestion and the various physiological functions of the human organism, are to be found in the vital foods and in the proper form to be appropriated in the process of nutrition. In other words, live on a more purely fruit and vegetable diet, eschew salt meats, soda biscuit, alum-made baker's bread, and especially lime water; or such as is found in the wells and springs contiguous to this city, and we might add all limestone regions, which often contain, in addition to lime, more or less magnesia.

We are well aware that this will antagonize existing customs, run counter to old established habit and prejudices, and meet with opposition from tradesmen and manufacturers; but if from observation and long experience we are satisfied of the danger to health and life that lurks in our present manner of living, and that immunity from pain, suffering and disease, with an existence made enjoyable, and life prolonged far beyond the usual time, and coming to an end as quietly and peacefully as a babe to its slumbers, will be brought about by this change, then it surely is our duty, and the demand is upon us to make the effort. Standing, as we do, in the great light which modern science has thrown on the causes of disease to the extent of completely revolutionizing our treatment, and lifting medicine from empiricism to the dignity of a true science, may we not as scientists continue our research till we are enabled to entirely prevent many of the incurables which now confront us? It is still a sad commentary on scientific medicine to acknowledge any disease as incurable, or without the pale of prevention. We acknowledge our skepticism regarding hereditary transmission of disease, believe in contagion as the more universal method of communication, and also that all diseases may be either cured or prevented.

Then, to repeat to some extent, besides Bright's disease, whose cause and fatality we believe may be found in calcareous deposits, we do know that we find these in the bladder as stone, and there are few physicians long in the practice but have witnessed the excruciating pain in the passage of a gallstone or renal calculus. A specimen of gallstone was presented to me a few years since, taken from a gall bladder which was full to complete impaction, its size larger than a goose egg. A physician informed me not long since that he counted more than three hundred of these stones in one evacuation from the bowels, and these he was told had been passing in like numbers in each evacuation for a week or more. Again, we see the arteries filling up with these deposits till they become rigid, brittle tubes. All these go to make up a list of dangerous and usually incurable diseases.

That this baneful influence may extend beyond the bounds of our present observation is within the limit of probability, but to what extent may never be demonstrated.

Now, if we are in fact just what we eat and drink, and we cannot be anything else, and many of our most serious maladies are the results of improper diet, can we not so regulate our food supply that each organ and system of organs will be supplied with nutrition just sufficient for the repair without any excess, and thus the functional harmony of each be maintained and carried on without trenching on the prerogative of another, and the whole of man's life, unmarred by pain or ache, be one of continued enjoyment till the measure of his days is full, when he passes into the realms of the great beyond, where disease has ever been a stranger and where pleasures flow continued as an everlasting river.—*Dental Headlight.*

